# Supplementary material for: Patterns of Intron Gain and Loss in Fungi
Source: PLoS Biol. 2004 Nov 30;2(12):e422. doi: 10.1371/journal.pbio.0020422 (PMC532390; doi:10.1371/journal.pbio.0020422)
Supplement: Table S1 — Also available at http://genes.mit.edu/NielsenEtAl/. (4.3 MB ZIP). [file pbio.0020422.st001.zip › NielsenEtAl/html/540.html]

AN6993.1.NCU05542.1.MG10052.1.FG08275.1


```
 CLUSTAL W (1.82) Multiple Sequence Alignments - Introns Inserted


Sequence 1: MG10052.1	205 aa
Sequence 2: FG08275.1	204 aa
Sequence 3: NCU05542.1	210 aa
Sequence 4: AN6993.1	207 aa
Alignment Length: 215 aa
Number Identitical Residues: 127 aa
Alignment Score (without introns) 5433


MG10052.1 	MVQKLYVKYND0VHKLCQESAPQILSDFQPQLMIAIGGGGYVPARILR~SFLKKPGNPNI
NCU05542.1	MVQKLYVTYND0VHNLCQEVAPKILETFNPQLMIAIGGGGYVPARILR~SFLKRPGSPNI
FG08275.1 	MVEKLYVTYND0VHKMCQKSAEKLLLDFQPQLLIAIGGGGYVPARILR~SFLKKGGAPNI
AN6993.1  	MPQKVYVTYNQ0VHKLCQSSAEQILNTFHPNLMIAIGGGGYVPARILR2SFLKRPGEPNI
          	* :*:**.**: **::**. * ::*  *:*:*:*************** ****: * ***

MG10052.1 	PIQAIGLSLYEQLSTDSVEEAPGTKVTRTQWLDLSSLGEMSNLLGKRILIVDEVDDTRTT
NCU05542.1	PIQAIGLSLYESLGED-VVEEPGTKVTRTQWLDLTALGSMKNLIGKRILIVDEVDDTRTT
FG08275.1 	PIQAIGLSLYESLGND-EVEAPGTKVTRTQWLDLTALGEMQNLVGKRILIVDEVDDTRTT
AN6993.1  	PIQAIGLSLYEDLGRGDPEEVPGTKVTRTQWLDLSSL-EMANLIGKNILIVDEVDDTRTT
          	***********.*. ..  * *************::* .* **:**.*************

MG10052.1 	LEYAVKELEKDVEAARVKAGYEGPKTEFQIFVLH0---NKNKNKKGTLPHEMLENGKYMA
NCU05542.1	LEYAVKELQKDVEAARQALGG-GEETQFAIFVLH0---NKDKPKKGTLPEDIISQGRYFA
FG08275.1 	LEYAVRELEKDVETARQKMGGTAPKTEFSIFVLH0---NKDKPKKGKLPDEMLAT-RYSA
AN6993.1  	LEYAVRELQKDVELAQKQLGREGEKTNFFVFVLH0LEQNKNKSKKGQLPTDMMESGRYHA
          	*****:**:**** *:   *  . :*:* :****  ..**:* *** ** :::  .:* *

MG10052.1 	ARTVEDVWICYPWEAA~DIDEHDRLASLQTKE-------
NCU05542.1	ALTVGDEWICYPWEAG~NIEEHDRNAAEAKAQKAAEAQN
FG08275.1 	AETVGDVWINYPWEAI~DIDEHDRNASLQTKSN------
AN6993.1  	AVTTDDVWICYPWEAK2DIDEHDALAKANPLV-------
          	* *. * ** *****  :*:***  *
```
